# Moxibustion for cervical vertigo: A protocol for a systematic review and meta-analysis

**DOI:** 10.1097/MD.0000000000021405

**Published:** 2020-07-31

**Authors:** Haiyan Li, Ting Yu, Pan Cheng, Siyu Qin, Lin Jiao, Rixin Chen

**Affiliations:** a Jiangxi University of Traditional Chinese Medicine; b Affiliated Hospital of Jiangxi University of Traditional Chinese Medicine, Nanchang, China.

**Keywords:** moxibustion, cervical vertigo, protocol, systematic review

## Abstract

**Background:**

This systematic review is designed to provide an assessment of the effectiveness and safety of moxibustion therapy for cervical vertigo (CV).

**Methods:**

Relevant randomized controlled trials (RCTs) will be searched from the databases of PubMed, the Cochrane Library, Embase, the China National Knowledge Infrastructure, Wanfang Database, Chinese Science and Technology Periodical Database, and Chinese Biomedical Literature Database from their inception to June 2020. Two authors will independently select studies, collect data, and assess the methodology quality by the Cochrane risk of bias tool. RevMan V.5.3 software will be used for statistical analysis.

**Results:**

The results of this study will provide an assessment of the current state of moxibustion for CV and aim to prove the effectiveness and safety of moxibustion therapy.

**Conclusion:**

This systematic review will provide a credible Evidence-based for the treatment of CV with moxibustion.

**Study registration number:**

INPLASY202060004.

## 1 Introduction

Vertigo is a common highly prevalent disease and one of the most common complaints in medical practice. Among them, cervical vertigo (CV) is a form of vertigo caused by cervical bone hyperplasia and cervical disc degeneration, which mainly manifests as dizziness, neck pain, blurred vision, and nausea.^[1–3]^ According to statistics, the incidence of CV in adults is about 10%, and it is more common in middle-aged and elderly people.^[4,5]^ With the change of people's living habits, the age of onset of CV tends to be younger, and the incidence rate gradually increases, which seriously affects the daily life of CV patients and also places a heavy burden on the health care system.^[6]^ In addition, the current treatment of CV is mainly based on drug treatment and surgical treatment. However, these treatments have limited efficacy and are often accompanied by certain adverse reactions.^[7]^

Moxibustion is an ancient external therapy and an important part of traditional Chinese medicine. In the past 2000, moxibustion has been widely used in clinical treatment in China. Moxibustion treatment usually involves the use of burning moxa sticks to generate heat and stimulate acupuncture points, which can relieve pain, relax muscles, and improve local tissue blood circulation.^[8,9]^ In addition, the theory of traditional Chinese medicine also believes that moxibustion has the effects of tonifying qi, activating blood, and dissolving stasis.^[8]^ Moxibustion is a noninvasive and easy-to-operate traditional treatment method with reliable efficacy and safety. Therefore, it is considered an effective alternative therapy.^[10,11]^

In recent years, moxibustion therapy has been widely used in clinic, and related clinical RCTs have proved that moxibustion is effective in treating CV.^[12]^ However, there is still a lack of systematic reviews on the effectiveness and safety of moxibustion for CV. Therefore, this systematic review will use the method of evidence-based medicine to analyze and evaluate the effectiveness and safety of clinical RCTs of moxibustion for CV, with a view to providing evidence-based evidence for further improving the clinical efficacy of CV patients.

## 2 Methods

### 2.1 Inclusion criteria for study selection

#### 2.1.1 Types of studies

RCTs assessing moxibustion treatment for CV will be eligible for inclusion. No language and publication status restrictions are there.

#### 2.1.2 Types of participants

CV patients with definite diagnosis will be included in this systematic review. There are no restrictions on race, age, sex, nationality, or disease severity. Participants who are not suitable for moxibustion will be excluded.

#### 2.1.3 Types of interventions

##### 2.1.3.1 Experimental interventions

Moxibustion therapy will include all therapies using any type of moxibustion, such as indirect moxibustion, direct moxibustion, heat-sensitive moxibustion, and so on. Mixed therapies based on moxibustion will also be included.

##### 2.1.3.2 Control interventions

The control group will receive one of the following treatment methods: conventional pharmacological therapy, no treatment, and placebo. RCTs comparing different types of moxibustion therapy will be excluded.

#### 2.1.4 Types of outcome measures.

##### 2.1.4.1 Primary outcome

Clinical efficacy, CV symptoms, and functional rating scale and visual analogue scale will be accepted as the primary outcomes.

##### 2.1.4.2 Additional outcomes

The safety assessment will be regarded as an additional result, which is judged according to the situation of adverse reactions.

### 2.2 Search methods for the identification of studies

The following electronic databases will be searched: PubMed, Embase, Cochrane Library, the China National Knowledge Infrastructure, Chinese Science and Technology Periodical Database, Wanfang Database, and Chinese Biomedical Literature Database. We will search the databases from the beginning to June 2020. Search terms consist of disease (CV, dizziness, cervical spondylopasis of the vertebroarterial type) and intervention (moxibustion OR moxa OR moxabustion OR mugwort) and research types (randomized controlled trial, controlled clinical trial, random trials). The PubMed search strategy is shown in Table 1.

**Table 1:**
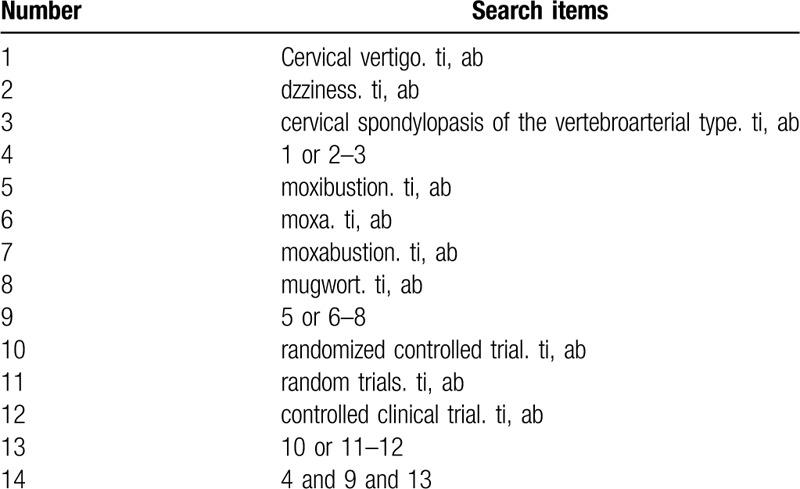
Search strategy used in PubMed database.

### 2.3 Data collection and analysis

#### 2.3.1 Selection of studies

**Figure 1:**
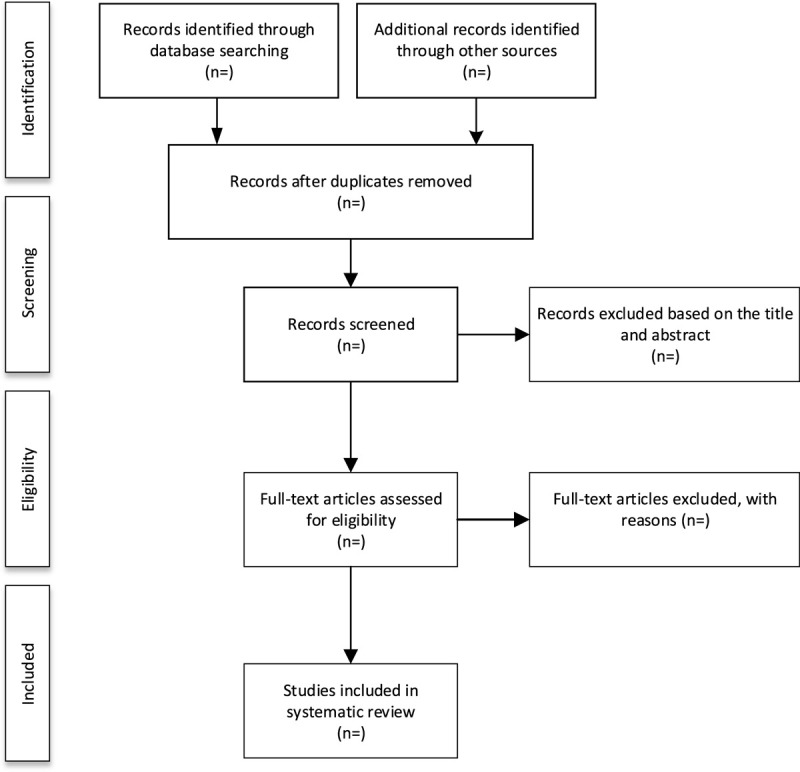
Flow diagram of study selection process.

We will import the retrieved results into EndNote X7 software and delete the duplicate data. After that, 2 authors will independently scan the titles, abstracts, and full texts of the literature according to the inclusion and exclusion criteria to evaluate the eligibility of these articles. Any different opinions will be resolved by the third author. The study selection procedure is summarized in Figure 1.

#### 2.3.2 Data extraction and management

Two reviewers will independently extract relevant data from the eligible RCTs, including the first author, participants’ baseline characteristics, sample size, intervention, intervention time, follow-up, results, and adverse events. Any discrepancies will be resolved through consultation with a third reviewer. If necessary, we will also contact the original author for more information.

### 2.4 Risk of bias assessment

The risk of bias (ROB) will be assessed by 2 authors based on the Cochrane collaboration's tool from 7 items: random sequence generation, allocation concealment, the blinding method for patients, researchers and outcomes assessors, incomplete result data, selective reports and other sources of bias. For each item, ROB will be graded as high, low, or unclear.^[13]^ Again, all disagreements will be resolved through discussion.

### 2.5 Quantitative data synthesis and statistical methods

#### 2.5.1 Quantitative data synthesis

We will use RevMan V.5.3 software for statistical analysis. Continuous variables will be calculated with mean difference and 95% confidence interval (CI). Categorical data will be calculated with the risk ratio and 95% CI.

#### 2.5.2 Assessment of heterogeneity

We will use *I*^*2*^ test and *χ*^2^ test to evaluate the heterogeneity of the results. When *I*^*2*^ ≤50% and *P* > .10, the results of the study will be considered as homogeneous; otherwise, it will be considered as heterogeneous.

#### 2.5.3 Assessment of reporting biases

Publication bias will be analyzed through the funnel plot. If the funnel plot is asymmetric, there may be a publication bias in the research results.

#### 2.5.4 Subgroup analysis

If significant heterogeneity is detected in our systematic review, we will perform subgroup analysis based on different control groups.

#### 2.5.5 Sensitivity analysis

When there are sufficient RCTs, we will conduct sensitivity analysis based on methodological quality, sample size, and missing data to evaluate the robustness of the research results.

#### 2.5.6 Grading the quality of evidence

The quality of evidence will be assessed based on the grading of recommendations assessment, development, and evaluation system, and divide the quality of evidence into 4 levels: high, medium, low, and very low.^[14,15]^

## 3 Discussion

Moxibustion is a traditional Chinese medicine external treatment method widely used in clinical treatment. Recent literature metrology analysis reports indicate that up to 364 diseases are suitable for treatment with moxibustion.^[16]^ Although the benefits of moxibustion for CV have been widely reported,^[17,18]^ the effectiveness of moxibustion has not been systematically and scientifically evaluated. The purpose of this systematic review is to evaluate the effectiveness and safety of moxibustion for CV. The conclusion of this study may provide evidence-based medical advice for moxibustion treatment of CV. However, this study may also have some potential limitations. First, during moxibustion treatment, there may be heterogeneity risks in acupoint selection, treatment time, and treatment frequency. Second, the measurements and tools of results of included RCTs may be different.

## Author contributions

**Data curation:** Haiyan Li, Ting Yu.

**Formal analysis:** Haiyan Li, Ting Yu.

**Investigation:** Ting Yu, Siyu Qin.

**Methodology:** Ting Yu, Pan Cheng.

**Project administration:** Haiyan Li, Lin Jiao.

**Software:** Ting Yu, Pan Cheng.

**Supervision:** Lin Jiao, Rixin Chen.

**Validation:** Lin Jiao, Pan Cheng.

**Visualization:** Siyu Qin, Pan Cheng.

**Writing – original draft:** Haiyan Li, Lin Jiao.

**Writing – review & editing:** Haiyan Li, Lin Jiao.
